# Supplementary material for: Coimmunization with Preerythrocytic Antigens alongside Circumsporozoite Protein Can Enhance Sterile Protection against Plasmodium Sporozoite Infection
Source: Microbiol Spectr. 2023 Feb 27;11(2):e03791-22. doi: 10.1128/spectrum.03791-22 (PMC10100930; doi:10.1128/spectrum.03791-22)
Supplement: Supplemental file 1 — Supplemental material. Download spectrum.03791-22-s0001.pdf, PDF file, 0.7 MB [file spectrum.03791-22-s0001.pdf]

Supplementary Material

Supplementary Tables

Supplementary Table 1. Individual challenge experiment outcomes

group	condition	Experiment (protected/total)						Total (protected/total)
		1	*2*	*3*	*4*	*5*	*6*	
A	gp120	0/5	0/5	0/5	0/5	0/5	0/5	0/30
B	gp120+PyCSP	1/5	1/5	2/5	0/5	1/5	1/5	6/30
C	PyHSP70-2+PyCSP		3/5		4/10		3/5	10/20
D	PyTRAP+PyCSP		3/5		4/10		3/5	10/20
E	PyCelTOS+PyCSP	4/5		2/5		2/5		8/15
F	PyP52+PyCSP		2/5	3/5		2/5		7/15
G	PyP36+PyCSP		1/5			1/5		2/10
H	PyGAMA+PyCSP	0/5					2/4	2/9
I	PySSP3+PyCSP	2/5				1/5		3/10
J	PyTRSP+PyCSP	2/5					2/5	4/10

Supplementary Figures

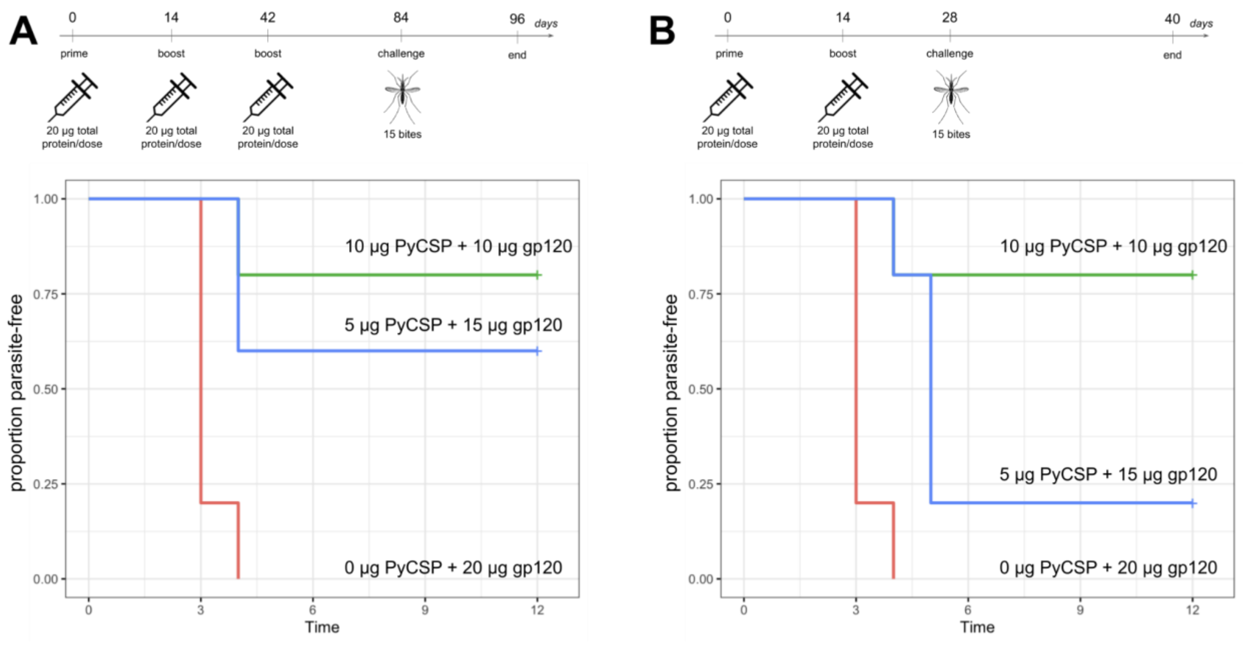

Supplementary Figure 1. Testing the CSP dose and immunization numbers to achieve the sub-optimal sterile protection.

Groups of mice (n=5) were immunized with the indicated amount of PyCSP per dose together with the gp120 control protein to achieve 20 µg of total protein content, injected in 20% Adjuplex. (**A**) shows a 3-dose regimen, followed by the challenge. (**B**) shows a 2-dose regimen, followed by the challenge.

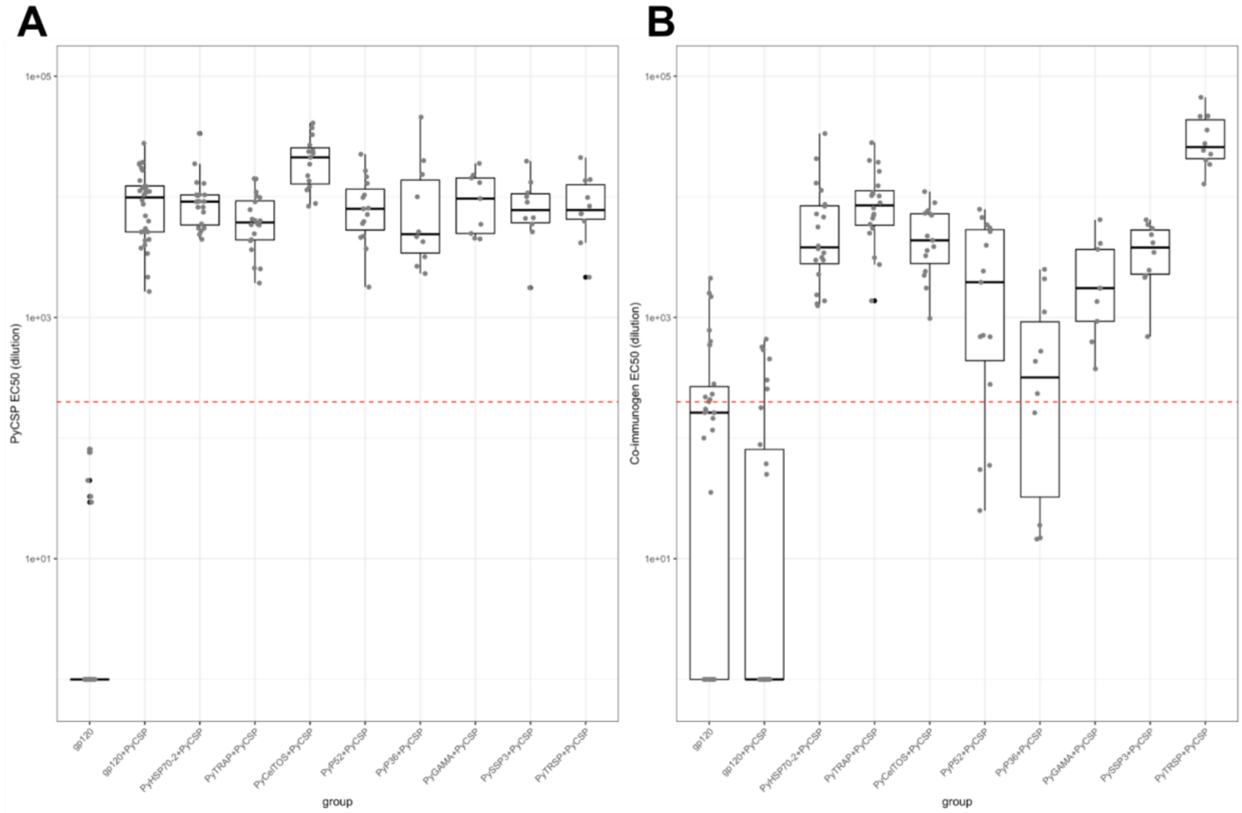

Supplementary Figure 2. Total immunoglobulin titers for immunized mice (for Figure 3).

EC50 values for PyCSP- (**A**) and co-immunogen-reactive immunoglobulins (**B**), shown in Figure 3, are plotted for each group. Each point represents a plasma sample; the conventional boxplot representation shows the per-group statistics including median, upper and lower quartile values. Red dashed line indicates the limit of detection (dilution of 1:200).

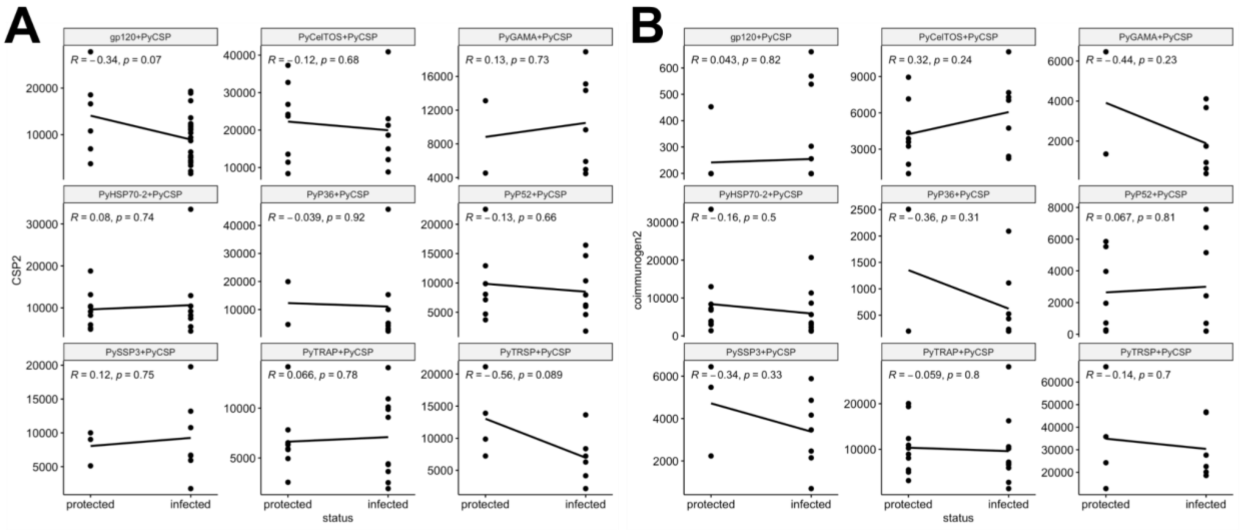

Supplementary Figure 3. Correlation analysis of the antibody titers with the status of protection.

Pearson correlation analysis against protection status using (**A**) the anti-PyCSP antibody titers (represented by EC50 values) or (**B**) the anti-co-immunogen antibody titers (represented by EC50 values).

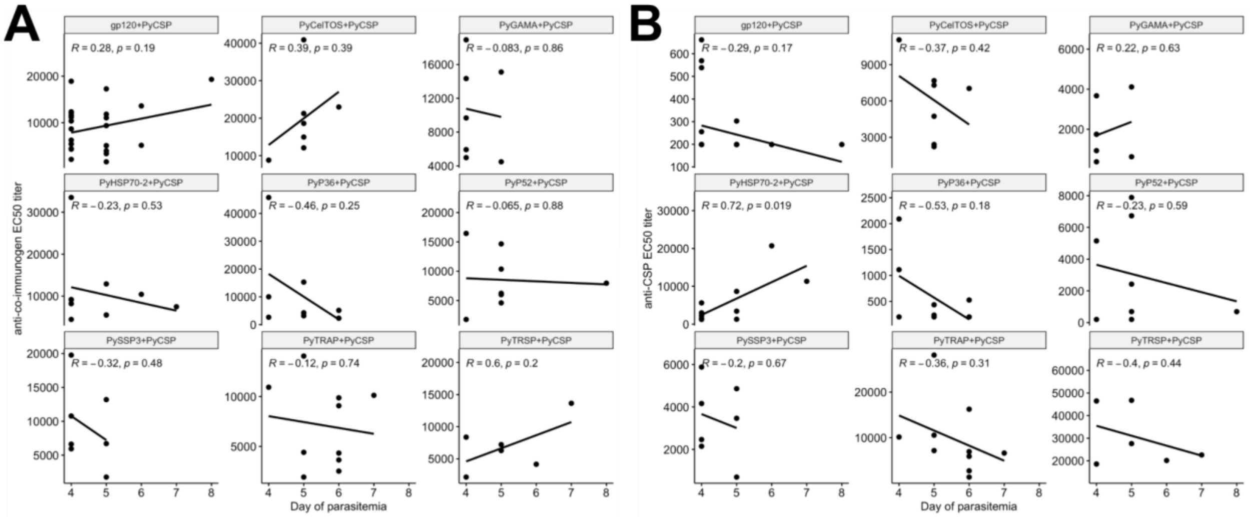

Supplementary Figure 4. Correlation analysis of the antibody titers with the first day of detectable parasitemia.

Pearson correlation analysis against the first day of detectable parasitemia using (**A**) the anti-PyCSP antibody titers (represented by EC50 values) or (**B**) the anti-co-immunogen antibody titers (represented by EC50 values).

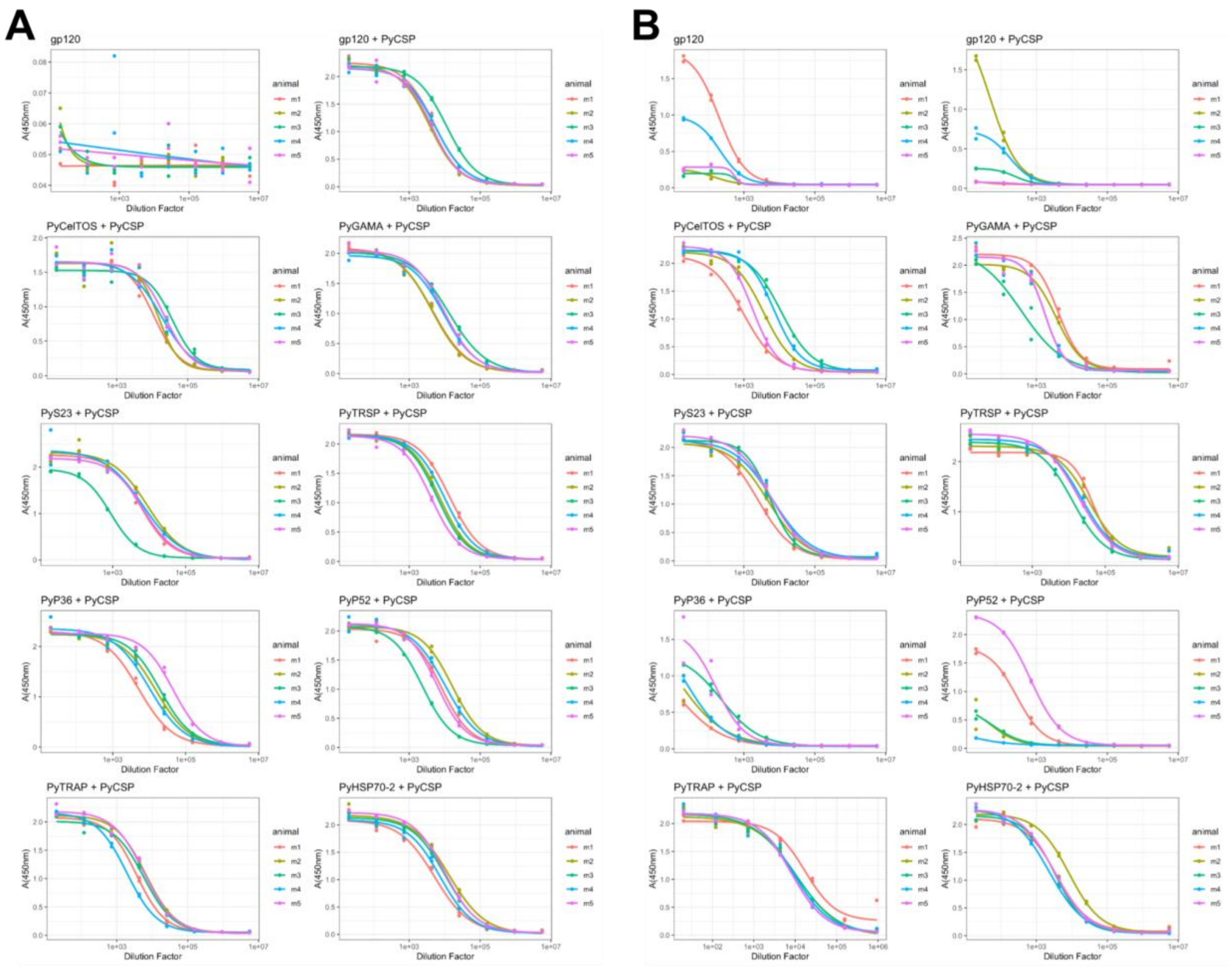

Supplementary Figure 5. Sample immunized-mouse plasma titration curves used for estimating the EC50 values.

Antigen-binding immunoglobulins in plasma samples from immunized mice were titrated using PyCSP (**A**) or the appropriate co-immunogen (**B**), measured in duplicate and shown as data points. Curve fits were constructed using the R package ‘drc’, shown as solid lines for each animal. Only a representative subset of data is shown here.
